# Modern Sociology

**Published:** 1902-10-18

**Authors:** 


### Modern Sociology.

### THE SOCIAL WORK OF THE SALVATION ARMY.

General Booth, who is now visiting Canada and the United States of America for the fourth time, is perhaps better known in connection with his religious than with his social work. He is associated in the average mind with somewhat noisy meetings at street corners, in which very earnest talk mingles curiously with exceedingly crude "music" and singing. To the General himself, there is probably no sweeter sound than this crude singing, and he has publicly said that he considers the bonnets of the "Salvation Lassies" very beautiful. But apart from religious methods on which a variety of opinion may exist, everyone must agree that the Social Scheme of the Salvation Army is an honest attempt to grapple with the problem of poverty and degradation in our great cities, nor can it be denied that a remarkable degree of success has attended the efforts made by the General in connection with his "Darkest England Scheme." The plan of work includes night shelters, prison gate homes, homes for the destitute of all ages, rescue and maternity homes and hospitals, elevators or factories, and a farm colony. Some of these we have visited under the guidance of an officer of the "Army," who most courteously showed us over the buildings and explained the methods of work.

The question as to whether common shelters which were not subject to the Common Lodging Houses Acts should be made so had been from time to time before the Public Health and Housing Committee of the London County Council before (as a result of legal proceedings) they became subject to that law. Some of the shelters belonging to the Salvation Army were previously inspected by the Medical Officer of Health, whose chief complaint was that an insufficient amount of cubic space was provided per head; in many cases it was, he stated, materially less than that required in common lodging houses in London. He added, however that things might have improved since his visit, and he was informed that it had been decided to confer with local sanitary authorities, with a view to arriving at an agreement as to the greatest number which should be admitted to any shelters in the district of the authority. With regard to the cleansing of the dormitories, the medical officer made no complaint; due care, he said, was taken to maintain the rooms in a state of cleanliness. The bedding (where beds were in use) was cleanly, and where bunks were used, the mattresses were taken out and swept daily, besides being periodically washed with a disinfecting fluid. The dormitories are generally large and lofty, but it must be remembered that in some cases old premises have been taken and converted to their present use, and that the best of a bad job has frequently to be made. The depressing effect of a visit late in the evening to one of these shelters is not due so much to the kind of accommodation provided, as to the hopelessness and dejection of the "lodgers." "The first thing we have to do," said the officer, "is to rouse hope in them." "Will they believe you when you speak of hope?" "No, but our system helps us. Men who are now in trusted positions can say, Look at me, I was as low as you are now." It is here that the essence of the scheme lies, and it is this that gives it its hold on the submerged population of our cities: it is on the plan of a moral object-lesson.

The same method is used in other departments of the work, and a writer on economics like Mr. Charles Booth (the statistician) and a State department like the Home Office bear record that the Salvation Army does touch and raise a class that has hitherto been the despair of ordinary organisations. "It is quite certain," says the Report of the Home Office Departmental Committee on Prisons, "that through their agencies a considerable number of apparently hopeless cases have been satisfactorily dealt with." Magistrates find that it is better to hand over certain classes of prisoners to the "Army" than to send them to a prison which cannot reform, but which does inflict lifelong disgrace; and this in itself is practical recognition.

In the Blackfriars Shelter we noticed a "text" on the wall: It was not necessary that any man who would work should commit suicide; and the work of finding employment for men and women rescued from starvation is a great one. We visited the Labour Exchange in Whitechapel Road and glanced through the books. A surprising number of respectable and even professional men find their way here; they have gone under through drink or ill-health, age, or mere want of moral backbone. A few doors off is a joiners' workshop, which turns out, along with carriages and furniture, the tambourines peculiar to the religious services of the "Army." This shop employs a large number of men. At Battersea we saw the extensive paper-making and tin works; in both cases the men sleep in a home where they lead a common life; they have a mess-room, smoking-room, dormitories and a common hall. An officer of the Army is in charge; he directs the work by day and conducts religious services in the evening. The men do not receive alms, but are paid by tokens, which represent money. They thus fare better and live more comfortably in proportion to the improvement in their work.

The farm colonists are drafted from the city elevators; there are some 260 of them. They come, mostly in weak health, knowing nothing of agriculture; their work for some months means a dead loss to the farm. Good food, however, and a regular life in the open air do wonders, and presently, under the beneficent influences of a healthy life' and human kindness, they improve. Self respect comes back, they are converted in another sense than that in current use in the Salvation Army, from disorder to order, from waste to work, from crime to honesty. They remain for three years; some are taken on as trained workmen and can train others, some are employed by friendly farmers, some go to the colonies abroad. There are failures, of course, but out of a hundred new arrivals about 55 per cent. are permanently restored to a self-respecting and self-dependent life. Allowing for waste, the farm still saves the country something like £20,000 a year, according to Sir Walter Besant's computation, apart from the moral gain which cannot be estimated in figures. The colonists at Hadleigh make their own bread, grow their own corn, fruit and vegetables, and it is proposed to buy for them a tea plantation in Ceylon. What is wanted now, to supplement the work at Hadleigh, is an "over-sea colony" in Australia or Canada.

Comparisons have been made between the Salvation Army scheme and the Poor Law system. "My great controversy with the Poor Law system," General Booth said recently at a Mansion House meeting, "is that it makes no attempt to change the character of the individual; after spending ten millions sterling the number of paupers is not reduced." Individual regeneration is the keynote of the Salvation Army's work; the unfortunate is treated as a friend "down on his luck," and as such he is helped, if he will allow himself to be helped, on to a higher plane. This is why, amid great discouragements and hindrances, the past ten years have seen such an astonishing success in the social scheme.

If figures mean anything, the following should be eloquent:—Altogether over 2,600 people are received in the elevators each year, and 873 ex-criminals are taken into the homes. Nearly 20,000 find employment, and 2,000 women are received into the Rescue Homes. A recent pamphlet states that the over-sea colony, alluded to above, is shortly to be established.

